# Association between Vessels Encapsulating Tumor Clusters and Circulating Tumor Cells in Hepatocellular Carcinoma: Clinical Evidence and Risk Model Development

**DOI:** 10.7150/ijms.111025

**Published:** 2025-06-12

**Authors:** Da-feng Xu, Rong-rong Li, Chang Shu, Ya-ni Li, Ran Tao, Yue-yue Chen, Hui-yuan Yang, Xiao-ping Chen, Jing-jing Yu, Wei Xiao

**Affiliations:** 1 Hepatic Surgery Center, Hubei Clinical Medicine Research Center of Hepatic Surgery and Hubei Key Laboratory of Hepato-Biliary-Pancreatic Diseases, Tongji Hospital, Tongji Medical College, Huazhong University of Science and Technology, Wuhan 430030, China.; 2 National Engineering Research Center for Nanomedicine, College of Life Science and Technology, Huazhong University of Science and Technology, Wuhan 430074, China.

**Keywords:** Hepatocellular carcinoma, VETC, Circulating tumor cells, Liver resection, Propensity score matching, prognostic prediction model

## Abstract

**Background:** Vessels encapsulating tumor clusters (VETC) and circulating tumor cells (CTCs) are recognized as emerging potential biomarkers in hepatocellular carcinoma (HCC), yet the underlying connection between them is not fully elucidated. This study aims to investigate the association between VETC and CTCs and evaluate their potential clinical utility.

**Methods:** This retrospective cohort study (NCT05297955) included 165 HCC patients who underwent curative hepatic resection. VETC was identified via CD34 immunohistochemical staining, and preoperative CTC levels were measured using the CellSearch platform. Propensity score matching (PSM) adjusted for confounders, and LASSO-Cox regression was used to develop a prognostic model.

**Results:** VETC-positive tumors were significantly associated with increased disease progression and shorter overall survival (OS) and disease-free survival (DFS). Elevated preoperative CTC counts showed a robust correlation with the VETC phenotype. The co-occurrence of VETC and CTCs emerged as a powerful prognostic indicator for both OS and DFS. A novel DFS prediction model, Vrisk, incorporating VETC, CTC, and four additional factors, demonstrated superior predictive performance compared to conventional staging systems.

**Conclusions:** The study establishes a strong association between VETC, elevated CTC levels, and poorer prognosis in HCC, providing critical insights into their functional roles and potential as biomarkers for clinical applications.

## Introduction

Hepatocellular carcinoma (HCC), the most common form of primary liver cancer, is the third leading cause of cancer death worldwide^1^. It primarily arises from chronic liver injury and inflammation due to viral hepatitis, alcohol consumption, or fatty liver disease^2^. Diagnosing and treating HCC is challenging because it often occurs late and progresses rapidly^3^.

In certain malignancies, such as Hepatocellular Carcinoma (HCC)^4^, tumor cells are encapsulated by a unique form of vascular architecture, referred to as Vessels Encapsulating Tumor Clusters (VETC). Once identified, it attracted significant attention because of its robust correlation with the prognosis of HCC patients^4^-^7^. A comprehensive multicenter study involving 541 HCC patients revealed that the presence of VETC is significantly associated with various clinical and pathological features, including elevated AFP levels, larger tumor size, poor differentiation, macrotrabecular pattern, reduced inflammatory infiltrates, and frequent MVI. Additionally, VETC is strongly correlated with early recurrence, disease-free survival, and overall survival^7^. VETC may also influence HCC treatment outcomes, as it has been found to predict the response to sorafenib and adjuvant TACE, two common treatments for advanced HCC^8^-^10^.

Although many studies have demonstrated that the presence of VETC in HCC is associated with a more aggressive disease phenotype, the precise underlying mechanisms remain incompletely understood. Current evidence suggests that VETC may promote HCC progression through multiple pathways. Primarily, the VETC pattern is associated with elevated intra-tumoral micro-vessel density (MVD) and larger tumor size in HCC, which may suggest a potential role in supporting tumor proliferation through enhanced vascular supply^11^. Additionally, VETC may enhance metastatic potential by facilitating hematogenous dissemination of malignant cells^4^. Emerging research also indicates that VETC could contribute to disease aggressiveness by modulating the tumor microenvironment, suppressing immune responses, and promoting tumor cell survival^12^, ^13^. For instance, recent studies have shown that VETC-positive HCC tumors exhibit molecular signatures associated with reduced immune activation, suggesting a potential role in immune evasion^14^, ^15^.

In various solid tumors such as breast and prostate cancer, circulating tumor cells (CTCs) are recognized as both biomarkers of disease progression and active mediators of metastasis^16^. As key drivers of cancer spread, CTCs exhibit unique biological traits that enhance their metastatic potential^17^. Their ability to enter and survive in circulation relies on specific adaptations, including resistance to anoikis, immune evasion, and tolerance to hemodynamic shear forces^18^. These mechanisms enable CTCs to withstand the hostile circulatory environment and promote distant metastasis across multiple cancer types^19^. In HCC, CTCs show emerging potential as prognostic indicators, though their clinical adoption requires further validation due to technical and biological heterogeneity^20^.

The shared characteristics of VETC and CTCs, such as their roles in tumor aggressiveness and metastasis, suggest a potential connection. Fang et al. reported that endothelium-covered tumor emboli were isolated from the bloodstream of VETC+ patients and tumor-bearing mice, suggesting that tumor cell clusters can enter the circulation through vascular anastomosis in an EMT-independent manner^4^. These findings align with the concept of CTCs and highlight a potential mechanism for CTC cluster formation in HCC. However, no further evidence has since emerged to clarify this relationship, leaving the hypothesis unexplored and highlighting the need for deeper investigation into their interplay. In this study, we conducted a retrospective cohort analysis that reveals a significant correlation among the VETC phenotype, elevated CTC levels, and adverse clinical outcomes in HCC patients. Building on these findings, we constructed a predictive model integrating these key biomarkers. The results offer valuable insights into the mechanistic role of VETC in driving HCC progression and highlight its utility as a promising biomarker for clinical applications, including prognosis assessment and treatment planning.

## Methods

### Patients

**Figure 1:**
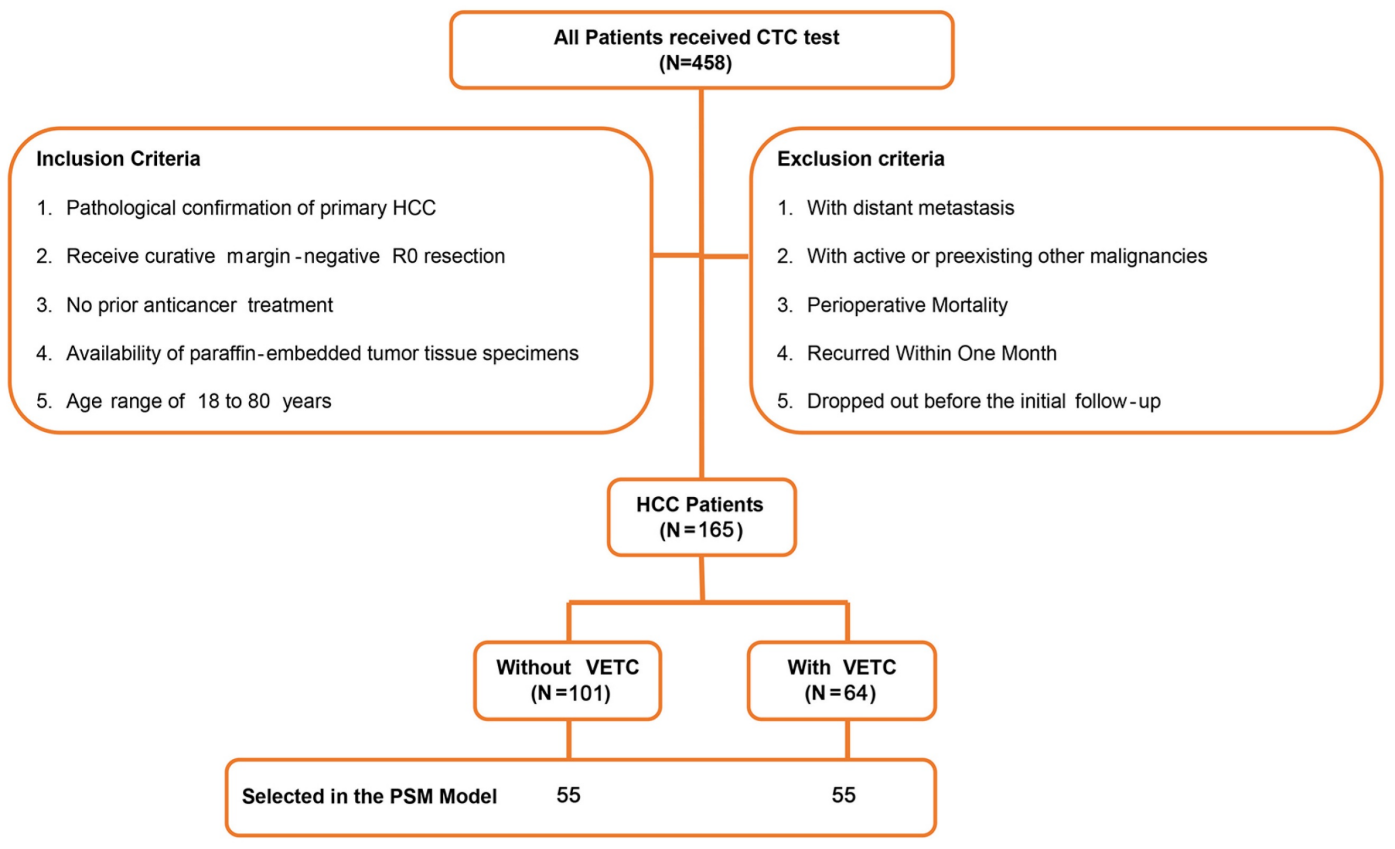
A flowchart represented the process of study design and patient distribution.

As previously described^21^, from December 2013 to August 2015, 458 individuals diagnosed with HCC and received liver resection were registered for CTC identification at the Hepatic Surgery Center, Tongji Hospital, Tongji Medical College, Huazhong University of Science and Technology. Of those, 165 patients were included in this retrospective study. The criteria for inclusion were: 1) pathological confirmation of primary HCC; 2) received curative treatment with margin-negative R0 resection; 3) no previous treatment for cancer; 4) the availability of paraffin-embedded tumor tissue specimens; and 5) age range of 18 to 80 years. The criteria for exclusion were: 1) detection of distant metastasis; 2) active or previous other types of cancer; 3) death during the perioperative period; 4) relapse within a two-month period; and 5) withdrawal prior to the initial follow-up. Figure 1 displayed a flowchart of the process of the study design and patient distribution.

Patients followed consistent surgical and oncological protocols. Follow-up was conducted regularly via phone calls and counterchecks. Overall survival (OS) was measured from surgery to death from any cause, and disease-free survival (DFS) from surgery to first recurrence, regardless of location. The average follow-up was 54.4 months. The study was approved by Tongji Hospital's ethics committee (TJ-IRB20211242), with informed consent waived, and retrospectively registered on ClinicalTrials.gov (NCT05297955).

### CTC analysis

Preoperative peripheral blood samples (7.5 mL) were collected into CellSave Preservative Tubes (Veridex, Janssen Diagnostics) one day before surgery. To minimize contamination from skin epithelial cells, an initial 5 mL blood sample was collected for parallel non-CTC biomarker testing prior to the assay tube. The samples were stored at room temperature and processed within 96 hours.

The CellSearch System (Veridex, Janssen Diagnostics) was employed for CTC isolation and enumeration. Peripheral blood cells were immunomagnetically enriched using ferrofluid nanoparticles coated with anti-EpCAM antibodies^21^, ^22^. Captured cells were fluorescently stained with anti-cytokeratin (CK 8, 18, 19) antibodies to identify epithelial origin, anti-CD45 antibodies to exclude leukocytes, and 4′,6-diamidino-2-phenylindole (DAPI) to confirm nuclear integrity. Cells meeting CTC criteria (intact morphology, CK+/DAPI+/CD45-) were identified and quantified using semiautomated fluorescence microscopy (CellTracks Analyzer).

Two independent analysts (JJ.Y. and W.X., co-authors of this study) evaluated the fluorescence images. Both researchers completed Veridex-certified CellSearch operator training and participated in quarterly inter-observer concordance assessments. Discrepancies in CTC counts (<5% of cases) were resolved through joint review with Veridex technical specialists, following the manufacturer's adjudication guidelines.

The 2 CTCs/7.5 mL cutoff was initially established in our prior study^21^, which utilized the same patient cohort. In brief, 139 patients were randomly divided into training (n=72) and validation (n=67) sets, with thresholds ranging from 1 to 10 CTCs systematically evaluated for overall survival (OS). The 2 CTCs threshold demonstrated the most significant prognostic discrimination in the training set (P < 0.05) and was validated in the independent validation set. Consistent with previous reports^23^, this threshold was further confirmed in the current study, reinforcing its clinical utility.

### Immunohistochemistry (IHC)

The VETC phenotype showed strong consistency between cancer tissue microarrays (TMAs) and whole-sectioned slides of HCC tissue specimens^7^. TMAs from HCC patients were used to assess CD34 expression through immunostaining under standardized conditions, as previously described^24^. The CD34 antibody (Proteintech, No. 14486-1-AP, dilution 1:1000) was employed for staining. VETCs were identified by distinct immunoreactivities forming continuous boundaries around tumor clusters. The VETC extent was semi-quantitatively measured as the proportion of VETC-positive areas relative to the total tissue section area (0%-100%). Samples were classified as VETC-positive if they exhibited the VETC phenotype and VETC-negative if they did not. Based on prior research^7^, a 55% cutoff was used to define the VETC phenotype: samples with ≥55% VETC coverage were categorized as VETC-high, while those with <55% coverage were categorized as VETC-low.

The macrotrabecular (MT) pattern was histologically defined by tumor trabeculae ≥6 cells thick in cross-section. Cases exhibiting MT morphology occupying ≥50% of the tumor area were classified as the macrotrabecular-massive (MTM) subtype^5^, ^7^.

### Statistical analysis

Fisher's exact test and chi-squared tests were used to evaluate proportional differences among groups. Mann-Whitney and Wilcoxon matched-pairs signed rank tests compared CTC counts across groups. Kaplan-Meier analysis and log-rank tests estimated and compared overall survival (OS) and disease-free survival (DFS). Univariate and multivariate Cox regression analyses identified factors associated with OS and DFS. Propensity score matching (PSM) was performed using the "MatchIt" package in R to address confounding factors, with variables including tumor number, size, microvascular/macrovascular invasion, BCLC stage, and TNM stage. VETC-positive and VETC-negative groups were matched 1:1 using nearest neighbor matching (caliper width: 0.02).

Given complex interrelationships among clinical parameters, LASSO regression was employed to construct a DFS prognostic model, leveraging its variable selection and regularization capabilities. All 13 clinical variables were included in the LASSO analysis using the "glmnet" R package. The model was developed in the training set and validated in the validation set, with stratified random sampling ("caret" package) ensuring balanced clinical parameter distribution. Statistical analyses were conducted using SPSS (v21.0) and R (v4.4.2), with significance set at P < 0.05 (two-sided). Data analysis and visualization were partially performed on the Hiplot Pro platform (https://hiplot.com.cn/).

## Results

### Clinicopathological characteristics of patients with VETC

**Figure 2:**
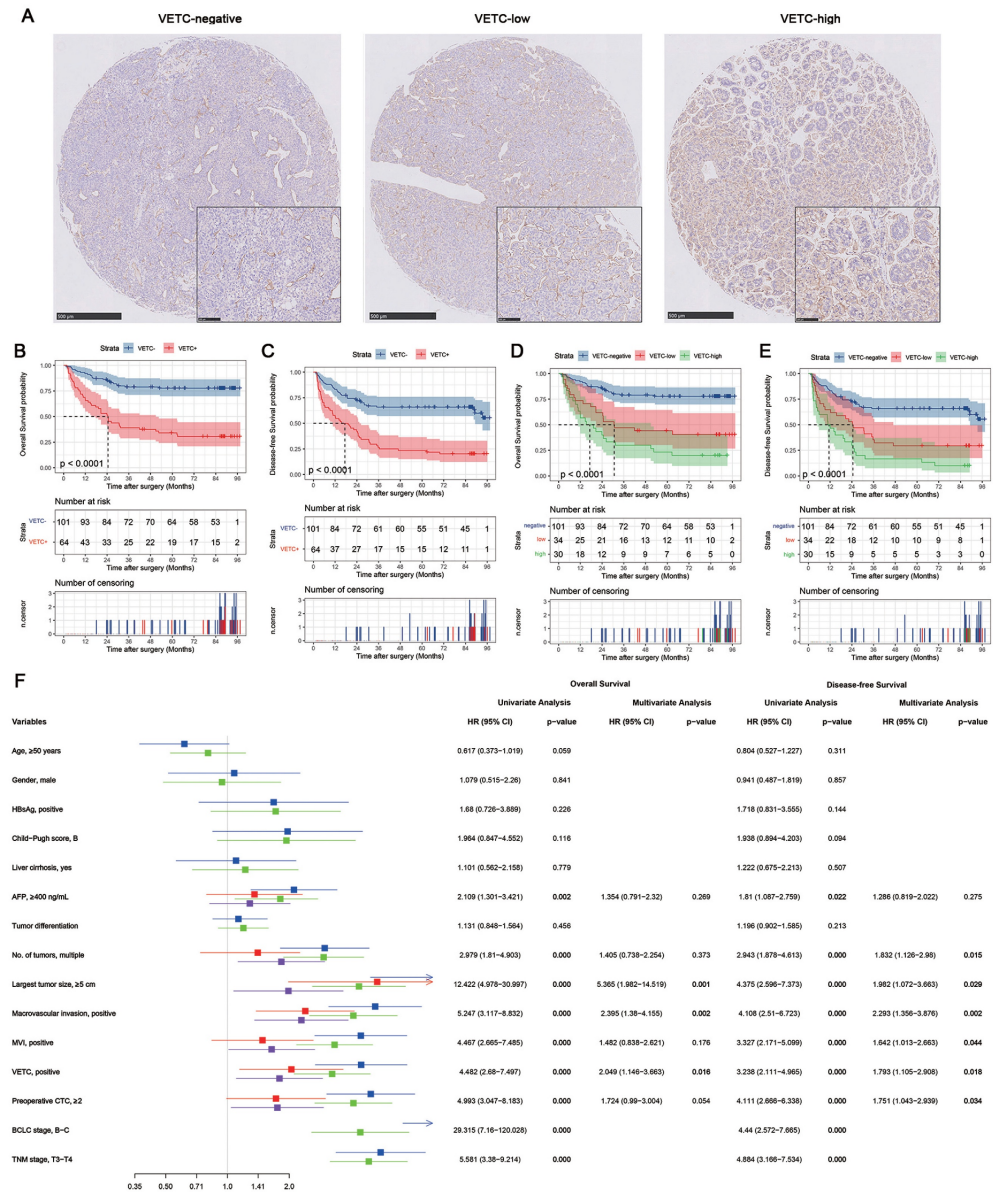
** The impact of the VETC phenotype on the survival of HCC patients.** (A) Representative images of Vessel encapsulating tumor clusters (VETC) in Hepatocellular carcinoma (HCC). The VETC phenotype was identified by the presence of CD-34 positive endothelial cells forming a continuous boundary around the tumor clusters. Tumor samples were categorized based on the extent of the VETC phenotype: those with VETC covering 55% or more of the tumor surface were classified as VETC-high, while samples with VETC covering less than 55% of the tumor surface were classified as VETC-low. (B) Kaplan-Meier curves represent the comparison of overall survival (OS) between patients with the VETC phenotype (VETC+, n=64) and those without the VETC phenotype (VETC-, n=101). (C) Comparison of disease-free survival (DFS) between VETC+ and VETC- patients. (D) Comparison of OS among patients with a higher extent of VETC (VETC-high, n=30), patients with a lower extent of VETC (VETC-low, n=34), and those without VETC (VETC-negative, n=101). (E) Comparison of DFS among VETC-high, VETC-low, and VETC-negative patients. (F) The forest plot presenting univariate and multivariate cox proportional regression analysis of factors associated with OS and DFS in full cohort of HCC patients.

Figure 2A shows representative IHC staining images of tissues classified as VETC-negative, VETC-low, and VETC-high. Table 1 outlines the demographic characteristics of the 165 HCC patients, stratified by VETC status. The cohort had a mean age of 49.9 years (range: 18-77), with 88.5% being male. Hepatitis B virus (HBV) infection was present in 87.3% of patients, and 83.6% had liver cirrhosis. Most patients (93.9%) had normal liver function (Child-Pugh score A), while 10 patients with Child-Pugh score B received preoperative liver protection. Based on the BCLC staging system, the cohort included 20 (12.1%) stage 0, 41 (24.8%) stage A, 76 (46.1%) stage B, and 28 (17.0%) stage C patients.

The presence of VETC in HCC tissues significantly correlated with tumor progression markers, including tumor number, larger tumor size, macrovascular invasion (MaVI), microvascular invasion (MVI), and higher BCLC and TNM stages (Supplementary Table S1). These associations strengthened with increasing VETC extents (Table 1). Notably, a subset of cases (22/165) exhibited macrotrabecular massive (MTM) patterns, with significant association with VETC.

### Prognostic value of VETC in HCC

The prognostic value of VETC was assessed using the log-rank test (Figure 2B-E). VETC-positive (VETC+) patients had significantly shorter overall survival (OS) (Figure 2B) and disease-free survival (DFS) (Figure 2C) compared to VETC-negative patients. Stratified analysis showed that in BCLC stage 0-A patients, VETC+ patients had numerically shorter OS (Supplementary Figure S1A) and DFS (Supplementary Figure S1B), but the differences were not statistically significant. In BCLC stage B-C patients, VETC+ patients exhibited significantly shorter OS (Supplementary Figure S1C) and DFS (Supplementary Figure S1D) compared to VETC-negative patients.

VETC-low patients had significantly shorter OS (Figure 2D, red vs. blue) and DFS (Figure 2E, red vs. blue) than VETC-negative patients. Similarly, VETC-high patients showed significantly shorter OS (Figure 2D, green vs. blue) and DFS (Figure 2E, green vs. blue) compared to VETC-negative patients. VETC-high patients also had numerically shorter OS (Figure 2C, green vs. red) and DFS (Figure 2D, green vs. red) than VETC-low patients, though the differences were less pronounced. Restricted cubic spline (RCS) analysis revealed a significant linear association between VETC extent (0%-100%) and DFS (p for overall < 0.001; p for nonlinearity = 0.251), while the association with OS was significant overall (p for overall < 0.001) but showed a trend toward nonlinearity (p for nonlinearity = 0.074). These results highlight VETC extent as a continuous predictor of both OS and DFS, with a stronger linear trend for DFS.

Cox proportional hazards regression was performed, comparing VETC-negative (VETC-) and VETC-positive (VETC+) groups due to small sample sizes in the VETC-low and VETC-high subgroups. Univariate analysis (Figure 2F) identified serum AFP levels, tumor number, tumor size, macrovascular invasion, microvascular invasion (MVI), BCLC stage, TNM-T stage, preoperative CTC, and VETC as significant factors for OS and DFS. Multivariate analysis, excluding BCLC and TNM stages to avoid potential confounding effects, revealed that tumor size, macrovascular invasion, preoperative CTC, and VETC phenotype were independent predictors of OS. Similarly, tumor number, tumor size, macrovascular invasion, MVI, preoperative CTC, and VETC phenotype were independent predictors of DFS. These findings underscore the prognostic significance of VETC and CTCs in HCC.

### Associations between VETC and CTC

Given the strong association between VETC and tumor-related characteristics, propensity score matching (PSM) was performed to minimize confounding factors between VETC+ and VETC- groups. In the PSM analysis, 55 VETC+ patients were matched 1:1 with VETC- patients. Demographic and clinical characteristics before and after PSM are detailed in Supplementary Table S1. Before PSM, no significant differences were observed in age, sex, HBsAg status, liver cirrhosis, Child-Pugh score, tumor differentiation, or serum AFP levels between the two groups. However, VETC+ patients had significantly or near-significantly higher tumor numbers, larger tumor sizes, more frequent macrovascular invasion, microvascular invasion (MVI), and advanced BCLC and TNM-T stages compared to VETC- patients. After PSM, these differences were effectively balanced, ensuring comparability between the groups.

**Figure 3:**
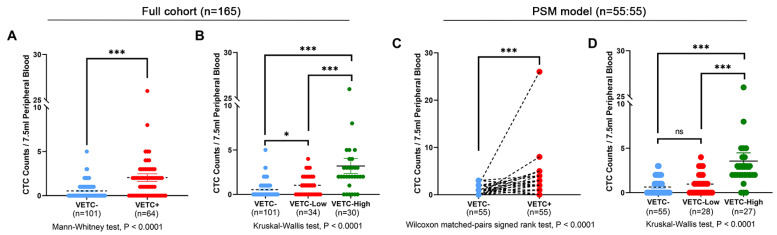
** Comparison of preoperative CTC counts among different VETC groups of HCC patients.** (A) Comparison of preoperative circulating tumor cell (CTC) counts between patients with the VETC phenotype (VETC+, n=64) and those without the VETC phenotype (VETC-, n=101) in full cohort of patients. Mann-Whitney test, P < 0.0001. (B) Comparison of preoperative CTC counts among VETC-high (n=30), VETC-low (n=34), and VETC-negative (n=101) groups in full cohort of patients. Kruskal-Wallis test, P < 0.0001. (C) Comparison of preoperative CTC counts between VETC+ (n=55) and VETC- (n=55) groups in patients of PSM model. Wilcoxon matched-pairs signed rank test, P < 0.0001. (D) Comparison of preoperative CTC counts among VETC-high (n=27), VETC-low (n=28), and VETC-negative (n=55) groups in patients of PSM model. Kruskal-Wallis test, P < 0.0001. *** = P < 0.001, * = P < 0.05, ns = not significant.

A significant correlation was found between the presence of VETC, and preoperative CTC counts in the entire cohort. Among VETC-negative patients, 12 out of 101 (11.9%) had two or more CTCs, whereas among VETC-positive patients, 34 out of 64 (53.1%) had two or more CTCs (Supplementary Table S1). The CTC count was significantly higher in the VETC-positive group compared to the VETC-negative group (Figure 3A). This association persisted after PSM: 11 out of 55 (20.0%) VETC-negative patients had two or more CTCs, while 30 out of 55 (54.5%) VETC-positive patients had two or more CTCs (Supplementary Table S1). Preoperative CTC counts remained significantly elevated in the VETC-positive group compared to the VETC-negative group (Figure 3C).

We further analyzed CTC counts in relation to varying degrees of VETC. In the full cohort, preoperative CTC counts significantly increased with greater VETC extent (Figure 3B). The VETC-high group had significantly higher CTC counts compared to both the VETC-low and VETC-negative groups. This trend was consistent in the PSM cohort, where the VETC-high group also showed significantly elevated CTC counts relative to the VETC-low and VETC-negative groups (Figure 3D). Supplementary Figure S3 illustrates the linear association between VETC extent and preoperative CTC counts, with Pearson correlation coefficients of 0.47 (p = 2.37e-10) in the full cohort (Supplementary Figure S3A) and 0.49 (p = 4.70e-08) in the PSM cohort (Supplementary Figure S3B), highlighting a strong positive correlation.

**Figure 4:**
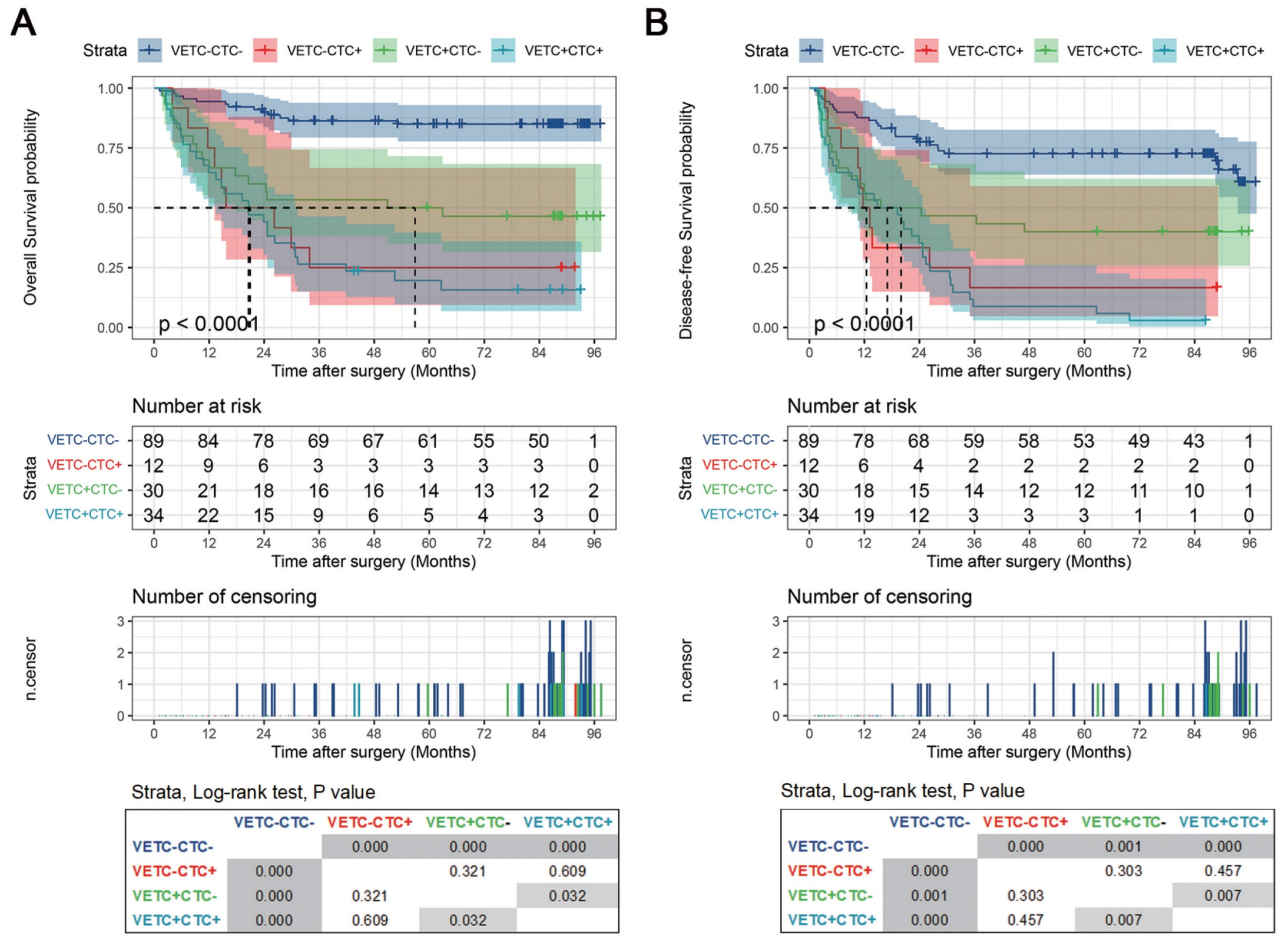
** Kaplan-Meier curves of stratified survival for VETC-CTC combination.** (A) Overall survival and (B) disease-free survival were compared using the log-rank test among patients with VETC+ and CTC ≥ 2 (VETC+CTC+, n=34), patients with VETC+ and CTC < 2 (VETC+CTC-, n=30), patients with VETC- and CTC ≥ 2 (VETC-CTC+, n=12), and patients with VETC- and CTC < 2 (VETC-CTC-, n=89). The results of pairwise log-rank test comparisons for each subgroup are displayed in the box at the bottom.

Next, we evaluated the clinical relevance of the VETC-CTC association. Pairwise comparisons of OS and DFS using the Log Rank (Mantel-Cox) test revealed significant differences among VETC-CTC subgroups. For OS (Figure 4A), the VETC-CTC- group had significantly better survival than the VETC-CTC+, VETC+CTC-, and VETC+CTC+ groups (all p < 0.001), with the VETC+CTC+ group showing the worst survival (p = 0.032 vs. VETC+CTC-). Similarly, for DFS (Figure 4B), the VETC-CTC- group had significantly better outcomes than the other groups (all p < 0.001 or p = 0.001), while the VETC+CTC+ group had significantly poorer DFS than the VETC+CTC- group (p = 0.007). These results demonstrate that combining VETC and CTC status effectively stratifies HCC patient outcomes, with the VETC+CTC+ subgroup associated with the worst prognosis for both OS and DFS.

### Vrisk, a VETC related prognostic prediction model

Finally, we developed the “Vrisk” prognostic model to predict disease-free survival (DFS) in HCC patients after curative surgery. The cohort of 165 patients was split into training and validation sets at a 7:3 ratio using stratified random sampling to ensure balanced distributions of clinical parameters (Supplementary Table S2). The LASSO Cox regression model identified six key predictors: tumor number, tumor size, macrovascular invasion (MaVI), microvascular invasion (MVI), VETC, and CTC (Supplementary Figure S4). Removing MaVI or VETC significantly reduced predictive accuracy, highlighting their critical roles, while CTC removal had a smaller impact (Supplementary Table S3). The model performed particularly well in BCLC stage B-C patients (C-index: 0.709), with MaVI and CTC being crucial for advanced-stage predictions.

**Figure 5:**
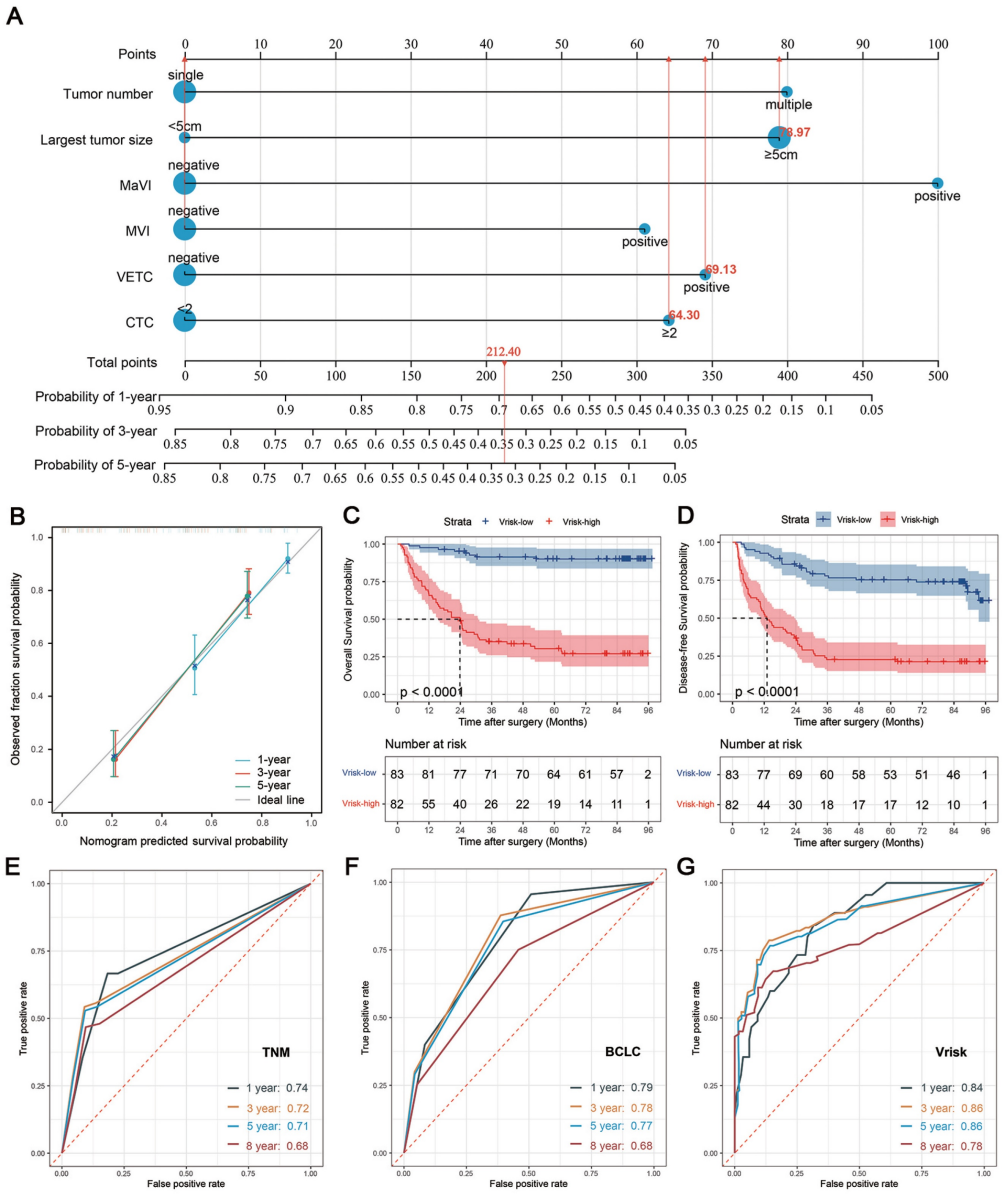
** Establishment of a prognostic model for predicting disease-free survival in HCC patients undergoing curative resection.** (A) Nomogram of the prognostic model, named "Vrisk," incorporating six tumor-related parameters (tumor number, largest tumor size, macrovascular invasion (MaVI), microvascular invasion (MVI), VETC and CTC). (B) Calibration curves of the nomogram. (C) Kaplan-Meier curves represent the comparison of overall survival (OS) between HCC patients in the full cohort with higher Vrisk score (Vrisk-high, n = 82) and those with lower Vrisk score (Vrisk-low, n = 83). The groups were divided into high- and low- risk categories using the median of the Vrisk score as the cut - off value. (D) Comparison of disease-free survival (DFS) between Vrisk-high and Vrisk-low patients in full cohort. (E-G) Time-dependent ROC curves at 1, 3, 5, and 8 years representing the predictive accuracy comparison among TNM staging (E), BCLC staging (F), and the "Vrisk" model (G).

A nomogram was created for clinical application, providing a user-friendly tool to estimate individual patient outcomes (Figure 5A). The Vrisk model achieved high predictive accuracy, with C-indices of 0.791 (training set), 0.759 (validation set), and 0.772 (overall cohort) (Supplementary Table S3). Calibration plots showed strong agreement between predicted and observed survival probabilities (Figure 5B). High-risk patients had significantly worse OS and DFS in the training set (Supplementary Figure S5A-B), validation set (Supplementary Figure S5C-D), and overall cohort (Figure 5C-D), demonstrating the model's robust discriminative ability.

Compared to traditional staging systems (BCLC and TNM), the Vrisk model consistently outperformed in predicting DFS, with higher C-indices across all cohorts (Supplementary Table S3). Time-dependent area under the receiver operating characteristic (tdAUROC) curves further validated its superiority: Vrisk achieved tdAUROC values of 0.84 (1 year), 0.86 (3 years), 0.86 (5 years), and 0.78 (8 years), outperforming TNM (0.74, 0.72, 0.71, 0.68) and BCLC (0.79, 0.78, 0.77, 0.68) staging systems (Figures 5E-G). These results underscore the Vrisk model's enhanced predictive performance for DFS in HCC patients.

## Discussion

Vessel-Encapsulating Tumor Clusters (VETC) and Circulating Tumor Cells (CTCs) are emerging potential biomarkers for disease progression and prognosis in hepatocellular carcinoma (HCC), supported by extensive research^5^-^7^, ^11^, ^25^-^30^. However, the direct relationship between these biomarkers remains underexplored. Beyond their shared association with disease progression, we hypothesize that the VETC structure promotes the generation and survival of CTCs for several reasons: First, early studies observed CTC clusters in VETC-positive tumor tissues, providing initial evidence of a potential link^4^. Second, the VETC architecture enhances blood supply to tumors, facilitating tumor cell intravasation into the vasculature^11^. Third, VETC enables metastasis independent of epithelial-mesenchymal transition (EMT)^4^, and CTCs detected by the CellSearch system are primarily epithelial-phenotype cells without EMT^31^. Finally, VETC induces an immunosuppressive tumor microenvironment^12^, ^32^, potentially aiding CTCs in evading immune surveillance. To investigate this hypothesis, we conducted a retrospective cohort study to explore the association between VETC and CTCs in HCC.

In this study, both VETC and CTCs consistently demonstrated significant prognostic value, as their presence in HCC patients was strongly associated with adverse outcomes across multiple survival analyses. The rigorous inclusion criteria and comprehensive 8-year follow-up period enhance the reliability of our findings. Notably, the patient cohort differed from most previous studies, with a predominance of intermediate and advanced-stage cases (BCLC B-C stage accounted for 63%), likely due to our institution being a regional referral center for complex cases. Our analysis revealed that VETC provided better prognostic discrimination in intermediate and advanced-stage HCC patients compared to early-stage cases, consistent with findings from another Chinese study^26^. Additionally, we identified a novel linear correlation between the extent of VETC (i.e., the proportion of VETC phenotype coverage in tumor samples) and patient prognosis, a finding not previously reported. This underscores the need for further standardization of VETC positivity thresholds in clinical practice. These results highlight the prognostic significance of VETC in advanced HCC patients, particularly those undergoing curative surgery.

Our cohort identified MTM in 13.3% (22/165) of HCCs, consistent with prior surgical series^5^, ^7^. Further analysis of MTM-related trends is provided in the Supplement Figure S6, MTM was associated with aggressive features (advanced stage, large size, vascular invasion, high AFP) and worse outcomes, aligning with its established prognostic role^33^, ^34^. Novelly, MTM tumors harbored higher CTC counts, with CTCs increasing alongside MT proportion, likely attributable to their vasculo-architectural properties facilitating tumor cell intravasation. However, near-complete MTM-VETC overlap (21/22 cases) precludes definitive isolation of MTM's independent contributions, necessitating future studies to disentangle their synergistic roles in dissemination.

One of the key findings of this study is the significant correlation between VETC and CTC in HCC. We validated this conclusion from several perspectives: Firstly, a significant association was observed between VETC presence and preoperative CTC counts in both the entire cohort and the propensity score-matched cohort. VETC-positive patients exhibited significantly higher CTC counts compared to VETC-negative patients, a trend that persisted after PSM. Secondly, CTC counts increased with greater VETC extent, and the VETC-high group consistently showed higher CTC counts than the VETC-low and VETC-negative groups. Finally, strong positive Pearson correlations further confirmed the linear association between VETC extent and CTC levels. These findings collectively offer direct clinical evidence supporting the association between VETC and CTCs, highlight VETC as a potential driver of CTC generation and dissemination in HCC.

We developed the Vrisk prognostic model to predict disease-free survival in HCC patients after curative surgery. Using LASSO Cox regression, six key variables were identified: tumor number, tumor size, macrovascular invasion (MaVI), microvascular invasion (MVI), VETC, and CTC. While CTC is a significant prognostic marker, its contribution to the Vrisk model was relatively limited, likely due to its correlation with other variables such as VETC and MaVI^35^. These correlations reduce CTC's incremental predictive value when combined with other factors. Nevertheless, CTC retains independent prognostic significance, supporting its role as a complementary biomarker in the Vrisk model. The simplified Vrisk model, excluding CTC, still maintains robust predictive performance, making it applicable in regions where CTC testing is unavailable or impractical.

Compared to traditional staging systems (e.g., TNM and BCLC), Vrisk consistently outperformed in predicting DFS, as demonstrated by higher C-indices and superior tdAUROC values. Notably, Lin et al. developed the VMNS model, which includes tumor number, tumor size, MVI, and VETC^36^. We compared the Vrisk model's predictive performance with a modified version that excluded MaVI and CTC (same parameters as the VMNS model but with different coefficients). The results showed that removing MaVI and CTC reduced Vrisk's predictive performance, particularly in advanced HCC patients. This discrepancy may stem from the predominance of advanced-stage patients in our cohort, emphasizing the importance of MaVI as a critical variable^37^. These findings highlight the robustness and clinical utility of the Vrisk model, especially for stratifying high-risk patients with advanced HCC.

The Vrisk model holds clinical translation potential. First, its discriminative capacity (tdAUROC 0.86 for 3-year DFS) facilitates precise risk stratification to tailor surveillance intervals: high-risk patients may require 3-month imaging versus 6-month for low-risk counterparts. Second, integrating VETC and MaVI identifies candidates for adjuvant therapy escalation. Third, the simplified model maintains robust performance (C-index 0.77 without CTC), offering a pragmatic solution for underserved regions.

This study has limitations. As a single-center retrospective analysis, it may be subject to selection biases inherent to observational designs. The model's external applicability is constrained by both the lack of independent validation cohorts and technical barriers: (1) the CellSearch platform—our CTC detection standard—has not been widely adopted in HCC research due to the high cost of equipment and testing, the lack of FDA and CFDA approval for HCC-specific applications, and the prolonged discontinuation of the service in China, all of which hindered collaboration across institutions and limited cross-institutional data sharing; (2) stringent inclusion criteria requiring paired VETC histology and CTC data substantially reduced eligible cases, though post-hoc power analysis confirmed sufficient sensitivity for key endpoints. To mitigate these limitations, we employed cross-validation with bootstrap resampling, demonstrating stable model performance. Future multicenter studies using harmonized protocols with emerging CTC detection technologies are warranted to validate these findings and enhance clinical translation in HCC management.

## Conclusion

In this retrospective cohort study, we analyzed 165 HCC patients who underwent curative surgery, evaluating VETC presence and extent via immunohistochemistry and comparing preoperative CTC counts across VETC phenotypes. Results revealed a significant positive correlation between VETC and preoperative CTC counts. VETC proved a robust prognostic marker, both independently and in combination with CTCs, emphasizing its role in HCC progression and metastasis. The Vrisk model, integrating VETC, CTC, and other key variables, demonstrated clinical utility in predicting disease-free survival and guiding postoperative management. These findings provide strong evidence supporting the VETC-CTCs association, offering valuable insights for HCC prognosis and treatment.

## Supplementary Material

Supplementary figures and tables.

## Figures and Tables

**Table 1:** Clinical Characteristics of HCC Patients and Correlation with VETC.

	VETC				
Variable	Overall (N=165)	Negative (N=101)	Low (N=34)	High (N=30)	p-value*
Age, years					0.051
≤ 50	90	53	15	22	
> 50	75	48	19	8	
Gender					0.512
Male	146	88	32	26	
Female	19	13	2	4	
HBsAg					0.375
Negative	21	12	3	6	
Positive	144	89	31	24	
Liver cirrhosis					0.177
No	27	19	2	6	
Yes	138	82	32	24	
Child-Pugh score					0.693
A	155	94	33	28	
B	10	7	1	2	
Largest tumor size, cm					0.000
≤ 5	66	52	12	2	
> 5	99	49	22	28	
No. of tumor					0.040
Single	127	83	26	18	
Multiple	38	18	8	12	
MVI					0.005
No	100	70	19	11	
Yes	65	31	15	19	
Macrovascular invasion					0.033
No	139	91	26	22	
Yes	26	10	8	8	
Tumor differentiation					0.438
Well	37	26	7	4	
Moderate	70	39	14	17	
Poor	58	36	13	9	
Macrotrabecular-massive					0.000
Negative	143	100	29	14	
Positive	22	1	5	16	
AFP, ng/mL					0.150
< 400	102	67	21	14	
≥ 400	63	34	13	16	
BCLC stage					0.000
0-A	61	50	10	1	
B-C	104	51	24	29	
TNM stage					0.002
T1-T2	113	78	22	13	
T3-T4	52	23	12	17	

* Pearson chi-square test, with Fisher's exact test used when expected frequencies < 5.

## Abbreviations

HCC: hepatocellular carcinoma
VETC: vessels encapsulating tumor clusters
AFP: α-fetoprotein
MVI: microvascular invasion
TACE: transcatheter arterial chemoembolization
MVD: micro-vessel density
CTCs: circulating tumor cells
EMT: epithelial-mesenchymal transition
EpCAM: epithelial cell adhesion molecule
BCLC: Barcelona Clinic Liver Cancer
TNM: Tumor Node Metastasis Classification of Malignant Tumors
PSM: propensity score matching
MaVI: macrovascular invasion
FDA: U.S. Food and Drug Administration
CFDA: China Food and Drug Administration
tdAUROC: Time-dependent area under the receiver operating characteristic
