# Supplementary material for: Association between Vessels Encapsulating Tumor Clusters and Circulating Tumor Cells in Hepatocellular Carcinoma: Clinical Evidence and Risk Model Development
Source: Int J Med Sci. 2025 Jun 12;22(12):2944–55. doi: 10.7150/ijms.111025 (PMC12243867; doi:10.7150/ijms.111025)
Supplement: Supplementary file 1 — Supplementary figures and tables. [file ijmsv22p2944s1.pdf]

Supplementary Materials

Table S1. Clinical characteristics of HCC patients before and after propensity score matching analysis.

Table S2. Clinical characteristics of HCC patients in the Training and Validation sets (7:3).

Table S3. Comparing the C-index of different models in HCC cohorts.

Figure S1. Kaplan-Meier curves illustrating the influence of the VETC phenotype on the survival of HCC patients stratified by BCLC stages.

Figure S2. Restricted Cubic Spline (RCS) analysis shows the relationship between the VETC phenotype and survival probability.

Figure S3. The scatter plot illustrates the linear association between the VETC phenotype and the preoperative CTC count.

Figure S4. Construction of the Vrisk prognostic prediction model using the Lasso Cox proportional hazards regression model.

Figure S5. Kaplan-Meier curves demonstrating the survival of HCC patients in the Training cohort and Validation cohort stratified by Vrisk.

Figure S6. Impact of macrotrabecular (MT) structures on clinicopathological features, survival outcomes, and CTC counts in HCC patients.

Table S1. Clinical characteristics of HCC patients before and after propensity score matching analysis.

Variable	All Patients VETC negative (N=101)	All Patients VETC positive (N=64)	p-value*	Patients in PSM Model VETC negative (N=55)	Patients in PSM Model VETC positive (N=55)	p-value*
Age, years			0.525			1.000
≤ 50	53	37		31	31	
> 50	48	27		24	24	
Gender			0.619			0.418
Male	88	58		45	49	
Female	13	6		10	6	
HBsAg			0.811			0.776
Negative	12	9		6	8	
Positive	89	55		49	47	
Liver cirrhosis			0.388			0.332
No	19	8		13	8	
Yes	82	56		42	47	
Child-Pugh score			0.742			0.363
A	94	61		51	54	
B	7	3		4	1	
AFP, ng/mL			0.142			0.696
Low (<400)	67	35		35	32	
High (≥400)	34	29		20	23	
No. of tumor			0.058			1.000
Single	83	44		39	39	
Multiple	18	20		16	16	
Largest tumor size,			**0.000**			1.000
≤ 5 cm	52	14		14	14	
>5 cm	49	50		41	41	
Macrovascular invasion			**0.015**			1.000
Negative	91	48		47	47	
Positive	10	16		8	8	
Tumor differentiation			0.335			0.606
Well	26	11		13	9	
Moderate	39	31		26	27	
Poor	36	22		16	19	
MVI			**0.005**			1.000
Negative	70	30		27	27	
Positive	31	34		28	28	
BCLC stage			**0.000**			1.000
0-A	50	11		11	11	
B+C	51	53		44	44	

**TNM stage**			**0.003**			1.000
**T1-T2**	78	35		35	35	
**T3-T4**	23	29		20	20	
**Preoperative CTCs**			**0.000**			**0.000**
**< 2**	89	30		44	25	
**≥ 2**	12	34		11	30	

* Pearson chi-square test, with Fisher's exact test used when expected frequencies < 5.

Table S2. Clinical characteristics of HCC patients in the Training and Validation sets (7:3).

Variable	Training set (N=116)	Validation set (N=49)	p-value*
Age, years			0.394
≤ 50	66	24	
> 50	50	25	
Gender			0.797
Male	103	43	
Female	13	6	
HBsAg			0.314
Negative	17	4	
Positive	99	45	
Liver cirrhosis			0.818
No	20	7	
Yes	96	42	
Child-Pugh score			0.725
A	108	47	
B	8	2	
AFP, ng/mL			0.862
Low (<400)	71	31	
High (≥400)	45	18	
No. of tumor			0.840
Single	90	37	
Multiple	26	12	
Largest tumor size,			1.000
≤ 5 cm	46	20	
>5 cm	70	29	
Macrovascular invasion			1.000
Negative	98	41	
Positive	18	8	
Tumor differentiation			0.996
Well	26	11	
Moderate	49	21	
Poor	41	17	
MVI			1.000
Negative	70	30	
Positive	46	19	
VETC			0.600
Negative	69	32	
Positive	47	17	
Preoperative CTCs			0.704
< 2	85	34	
≥ 2	31	15	

**BCLC stage**			1.000
**0-A**	43	18	
**B+C**	73	31	
**TNM stage**			0.856
**T1-T2**	80	33	
**T3-T4**	36	16	

* Pearson chi-square test, with Fisher's exact test used when expected frequencies < 5.

Table S3. Comparing the C-index of different models in HCC cohorts.

Model	Training set	Validation set	Overall
	C-index (95%CL)		
TNM stage	0.685 (0.627-0.743)	0.661 (0.570-0.752)	0.675 (0.627-0.723)
BCLC stage	0.721 (0.665-0.778)	0.712 (0.638-0.787)	0.714 (0.669-0.759)
Vrisk model	0.791 (0.739-0.842)	0.759 (0.687-0.831)	0.772 (0.729-0.815)
**Effect of deleting variable from Vrisk model**			
Vrisk - VETC	0.780 (0.723-0.837)	0.745 (0.671-0.818)	0.757 (0.712-0.802)
Vrisk - CTC	0.789 (0.734-0.843)	0.752 (0.674-0.829)	0.770 (0.727-0.813)
Vrisk - MaVI	0.777 (0.725-0.830)	0.760 (0.683-0.837)	0.763 (0.719-0.806)
Vrisk – VETC & CTC	0.777 (0.722-0.832)	0.728 (0.651-0.804)	0.754 (0.710-0.799)
Vrisk – VETC & MaVI	0.763 (0.709-0.817)	0.742 (0.672-0.812)	0.742 (0.699-0.785)
Vrisk – MaVI & CTC	0.775 (0.724-0.827)	0.746 (0.666-0.827)	0.758 (0.715-0.802)
**Model**	**BCLC stage 0-A**	**BCLC stage B-C**	**Overall**
	**C-index (95%CL)**		
Vrisk model	0.637 (0.516-0.757)	0.709 (0.641-0.777)	0.772 (0.729-0.815)
Vrisk - MaVI&CTC	0.636 (0.514-0.759)	0.674 (0.606-0.742)	0.738 (0.705-0.802)

VETC, vessels encapsulating tumor clusters; MaVI, macrovascular invasion; CTC, circulating tumor cell.

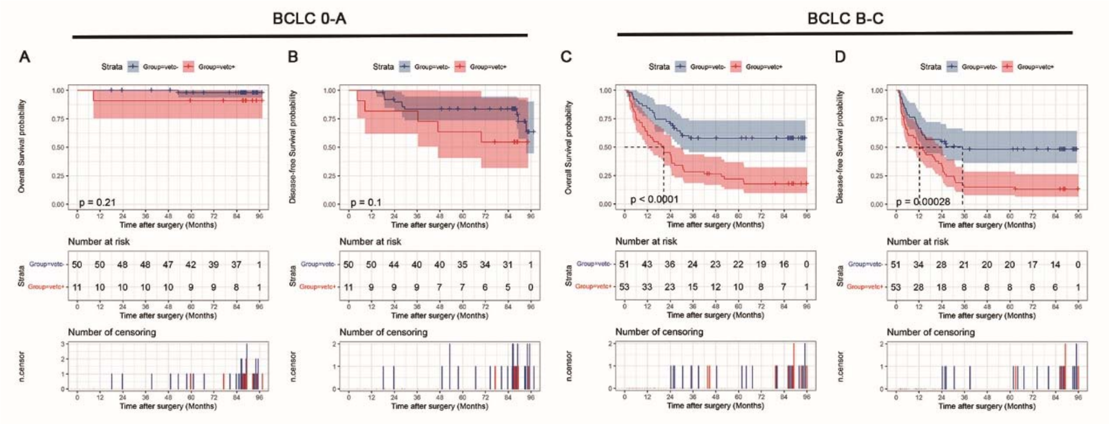

Figure S1. Kaplan-Meier curves illustrating the influence of the VETC phenotype on the survival of HCC patients stratified by BCLC stages.

(A) Comparison of overall survival (OS) between HCC patients at BCLC stage 0-A with the VETC phenotype (VETC+, n = 11) and those without the VETC phenotype (VETC-, n = 50).

(B) Comparison of disease-free survival (DFS) between VETC+ and VETC- patients at BCLC stage 0-A.

(C) Comparison of overall survival (OS) between HCC patients at BCLC stage B-C with the VETC phenotype (VETC+, n = 53) and those without the VETC phenotype (VETC-, n = 51).

(D) Comparison of disease-free survival (DFS) between VETC+ and VETC- patients at BCLC stage B-C.

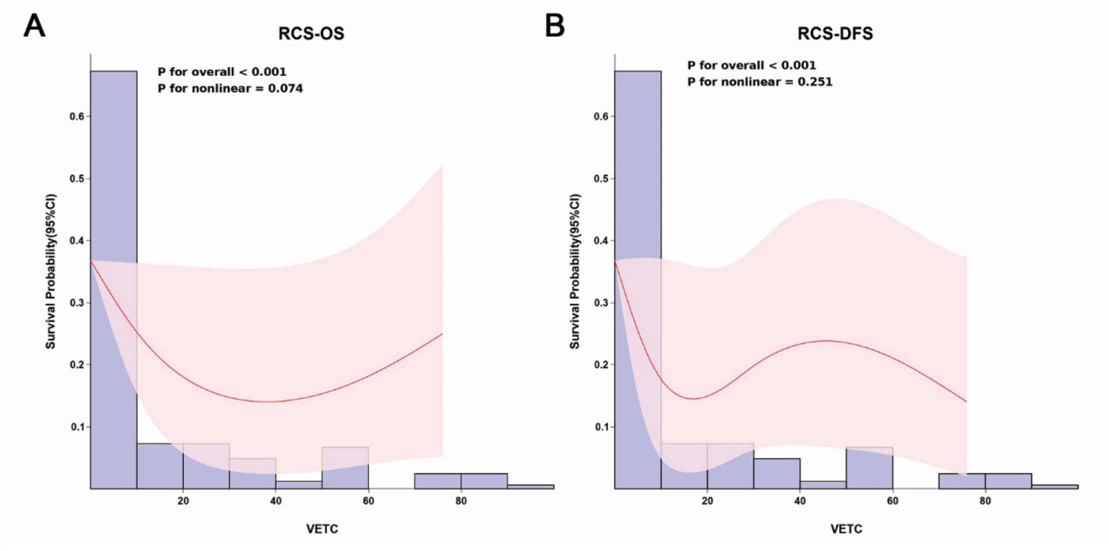

Figure S2. Restricted Cubic Spline (RCS) analysis shows the relationship between the VETC phenotype and survival probability.

The blue bars represent the VETC level, and the pink shaded area represents the confidence interval. The P - values indicate significant differences:

(A) The association between the VETC level and Overall survival probability (95% CI). P for overall < 0.001 and P for nonlinear = 0.074.

(B) The association between the VETC level and disease - free survival probability (95% CI). P for overall < 0.001 and P for nonlinear = 0.251.

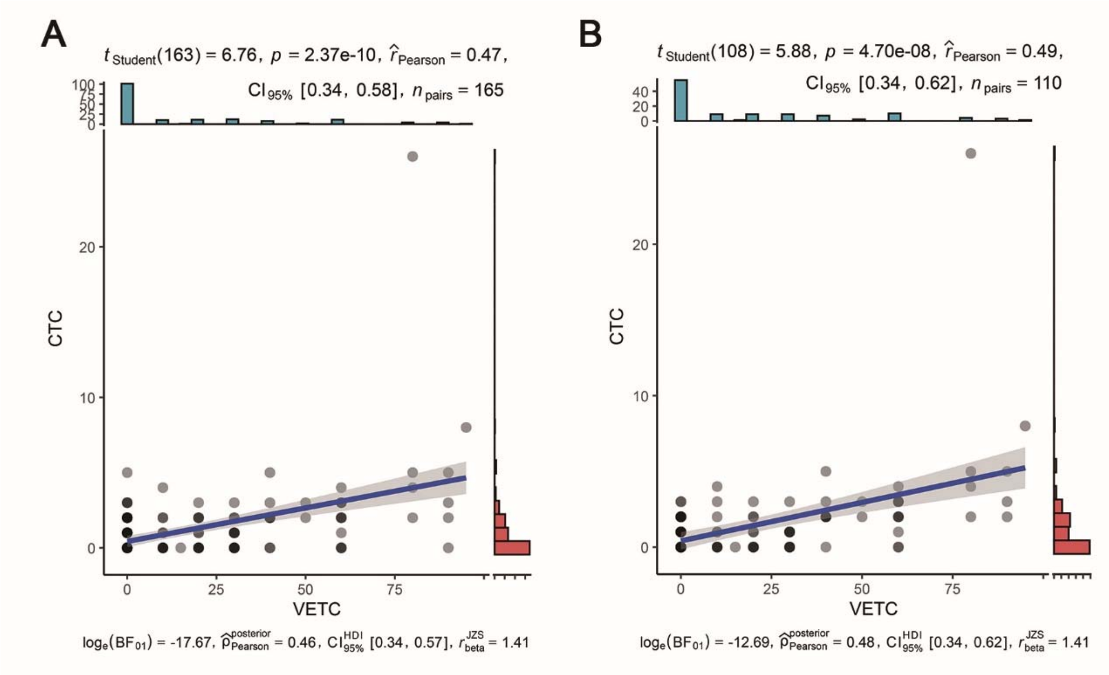

Figure S3. The scatter plot illustrates the linear association between the VETC phenotype and the preoperative CTC count.

The red bars and blue bars display the distributions of CTC count (ranging from 0 to 26) and VETC (ranging from 0 to 100%), respectively. Each scatter point represents the condition of a single case. The Pearson analysis is used for correlation analysis.

(A) The data shown represents the overall HCC patient cohort (n = 165).

(B) The data shown represents the HCC patient cohort in the PSM model (n = 110).

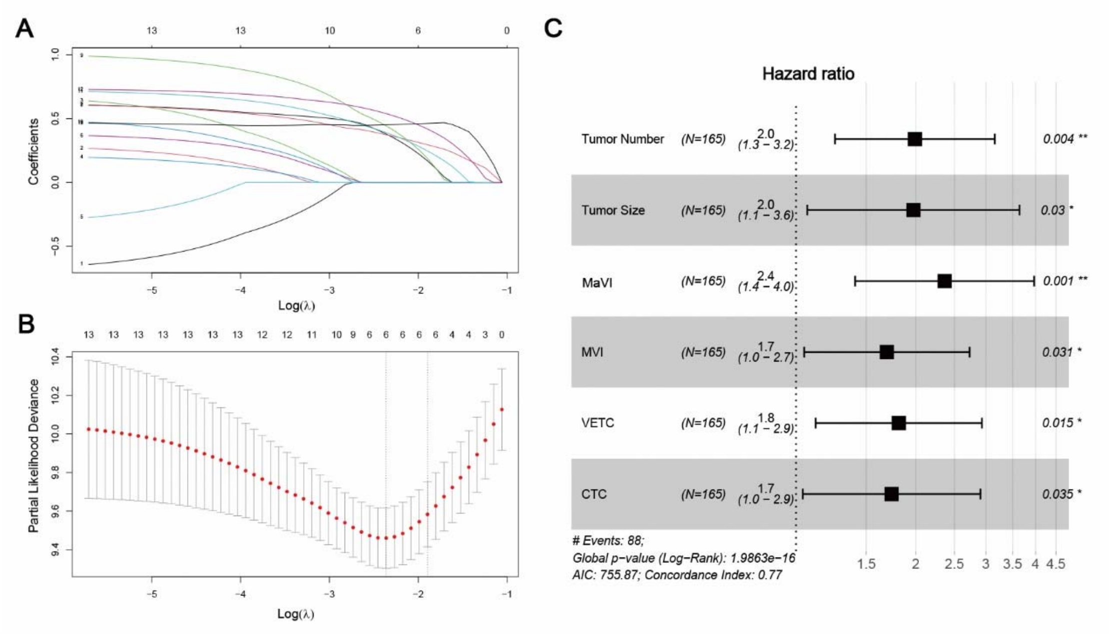

Figure S4. Construction of the Vrisk prognostic prediction model using the Lasso Cox proportional hazards regression model.

(A) Depicts the dynamic changes of LASSO coefficients for 13 variables during the iterative model construction process. Each line represents the coefficient trajectory of a variable incorporated into the Vrisk prognostic prediction model via Lasso Cox proportional hazards regression.

(B) The two vertical lines are placed at optimal points from the minimum and 1 - SE criteria. The minimum criteria select 6 variables (tumor number, tumor size, MaVI, MVI, VETC and CTC) strongly associated with the model outcome.

(C) The forest plot presents the final model variables. It shows the selected 6 variables along with their corresponding coefficients, hazard ratios and the p-values in the multivariate Cox regression analysis.

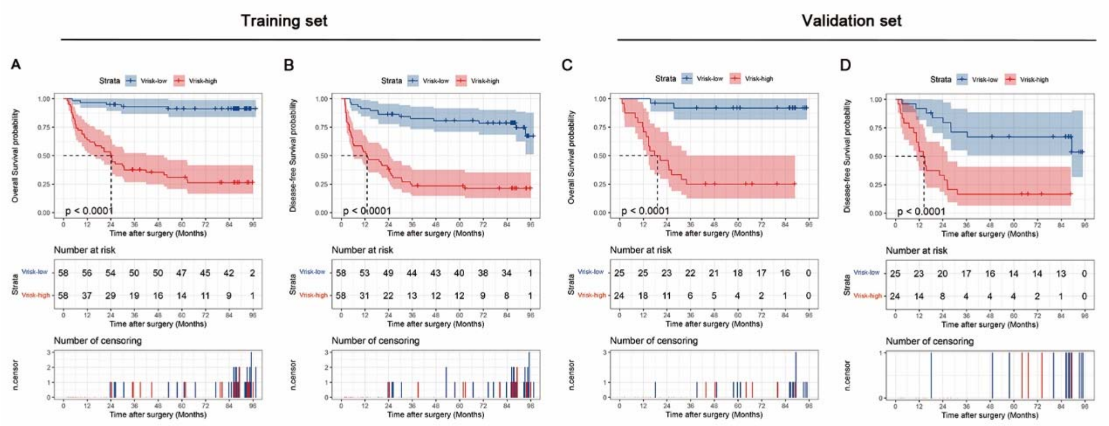

Figure S5. Kaplan-Meier curves demonstrating the survival of HCC patients in the Training cohort and Validation cohort stratified by Vrisk.

Risk groups were determined based on the proposed Vrisk score, with the groups being divided into high- and low- risk categories using the median of the Vrisk score as the cut-off value.

(A) Comparison of overall survival (OS) between HCC patients in the training cohort with higher Vrisk score (Vrisk-high, n = 58) and those with lower Vrisk score (Vrisk-low, n = 58).

(B) Comparison of disease-free survival (DFS) between Vrisk-high and Vrisk-low patients in the training cohort.

(C) Comparison of overall survival (OS) between HCC patients in the validation cohort with higher Vrisk score (Vrisk-high, n = 24) and those with lower Vrisk score (Vrisk-low, n = 25).

(D) Comparison of disease-free survival (DFS) between Vrisk-high and Vrisk-low patients in the validation cohort.

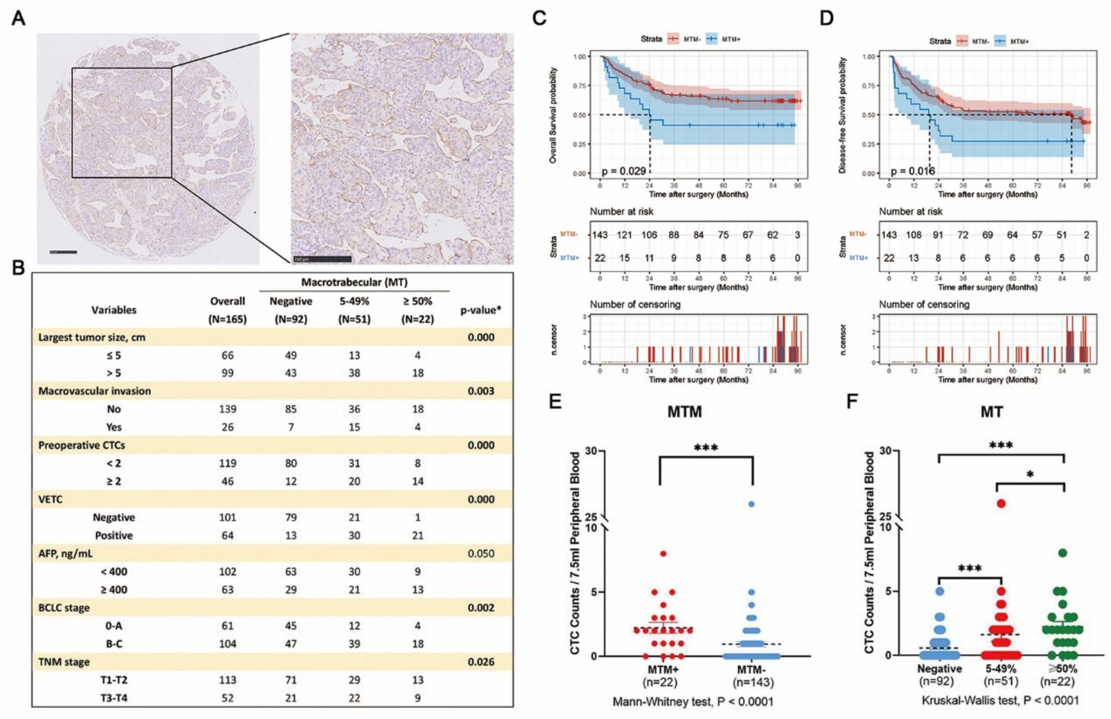

Variables	Overall (N=165)	Macrotrabecular (MT) Negative (N=92)	5-49% (N=51)	≥ 50% (N=22)	p-value*
Largest tumor size, cm					0.000
≤ 5	66	49	13	4	
> 5	99	43	38	18	
Macrovascular invasion					0.003
No	139	85	36	18	
Yes	26	7	15	4	
Preoperative CTCs					0.000
< 2	119	80	31	8	
≥ 2	46	12	20	14	
VETC					0.000
Negative	101	79	21	1	
Positive	64	13	30	21	
AFP, ng/mL					0.050
< 400	102	63	30	9	
≥ 400	63	29	21	13	
BCLC stage					0.002
0-A	61	45	12	4	
B-C	104	47	39	18	
TNM stage					0.026
T1-T2	113	71	29	13	
T3-T4	52	21	22	9	

Figure S6. Impact of macrotrabecular (MT) structures on clinicopathological features, survival outcomes, and CTC counts in HCC patients.

(A) Representative immunohistochemical staining of macrotrabecular (MT) structures in HCC tissue.

(B) Correlation between MT structures and clinicopathological features in HCC patients. Data are presented as numbers of patients. Statistical significance was determined using the chi-square test (p-value).

(C) Kaplan-Meier survival curves for overall survival (OS) stratified by MTM status. Patients with MTM-positive tumors had significantly worse OS compared to those with MTM-negative tumors (log-rank test, p = 0.029).

(D) Kaplan-Meier survival curves for disease-free survival (DFS) stratified by MTM status. Patients with MTM-positive tumors had significantly worse DFS compared to those with MTM-negative tumors (log-rank test, p = 0.018).

(E) Comparison of CTC counts in peripheral blood between MTM-positive and MTM-negative HCC patients. CTC counts were significantly higher in MTM-positive patients (Mann-Whitney U test, p < 0.0001).

(F) Distribution of CTC counts in peripheral blood according to the percentage of MT structures in HCC tumors. CTC counts increased with the proportion of MT

structures (Kruskal-Wallis test, $p < 0.0001$).
